# Supplementary material for: A tradeoff between enterovirus A71 particle stability and cell entry
Source: Nat Commun. 2023 Nov 17;14:7450. doi: 10.1038/s41467-023-43029-0 (PMC10656440; doi:10.1038/s41467-023-43029-0)
Supplement: Supplementary file 1 — Supplementary Information [file 41467_2023_43029_MOESM1_ESM.pdf]

Supplementary Materials:

A tradeoff between enterovirus A71 particle stability and cell entry

Adam Catching[1,2], Ming Te Yeh[1], Simone Bianco[3], Sara Capponi[4,5*], Raul Andino[1,*]

[1] Department of Microbiology and Immunology, University of California, San Francisco, San Francisco, CA 94158

[2] Graduate Program in Biophysics, University of California, San Francisco, San Francisco, CA 94158

[3] Altos Labs, Los Altos, CA 94022

[4] Industrial and Applied Genomics, AI and Cognitive Software, IBM Almaden Research Center, San Jose, CA, 95120

[5] Center for Cellular Construction, San Francisco, CA 94158

[*] corresponding authors: sara.capponi@ibm.com; raul.andino@ucsf.edu

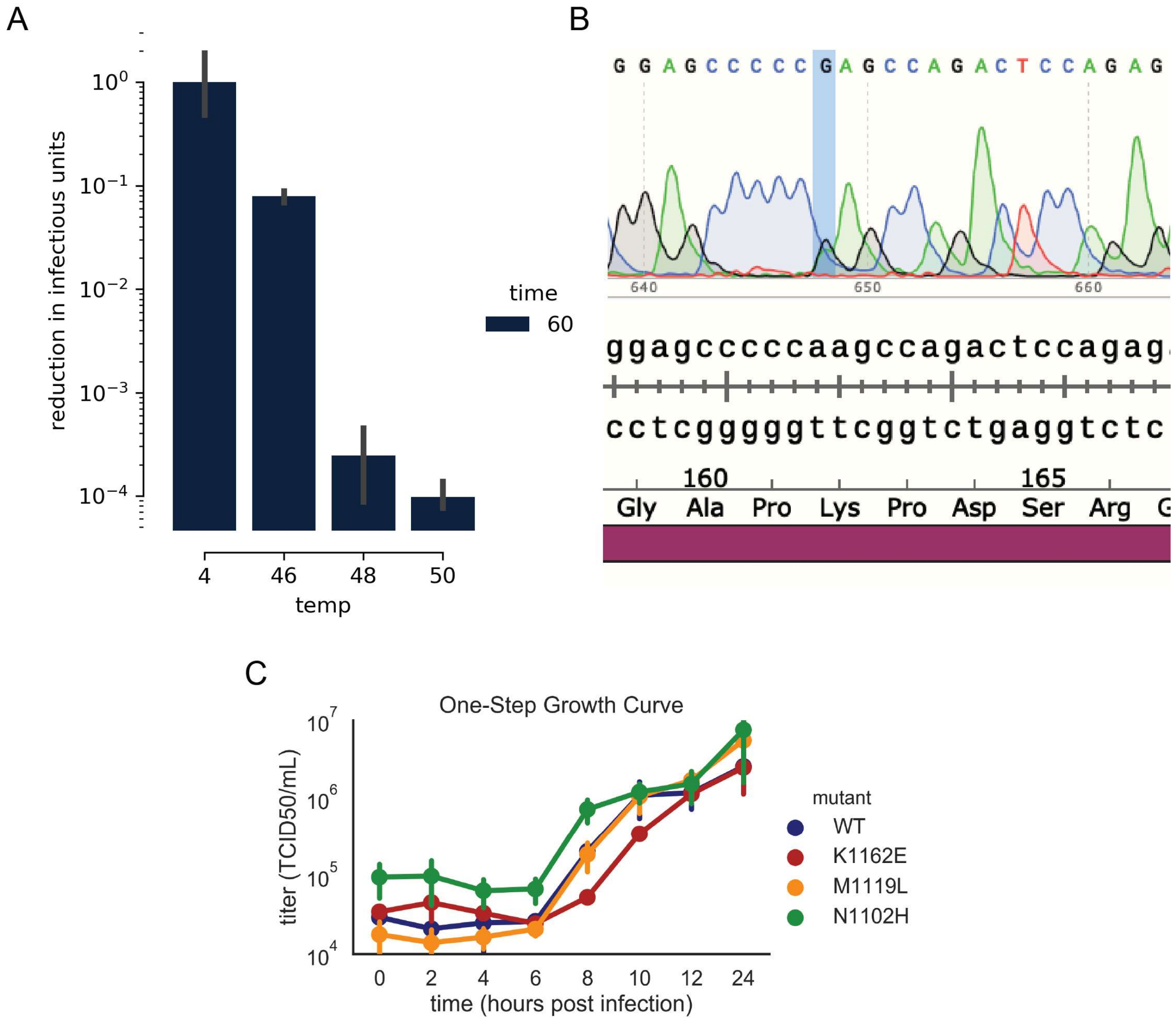

Fig. 1. Optimization of temperature selection, identification and characterization of thermostable variants. A) Optimization of temperature selection. Wild-type (WT) EV-A71 was subjected to different temperatures for 1 hour. The degree of virus inactivation was determined by tissue-culture infectious dose 50 ($TCID_{50}$) in RD cells. B) Sequencing analysis of heat-selected virus at passage 6. A->G transition observed at position 648 of VP1 coding region. C) One-step growth curve of viruses carrying mutations identified in treated populations.

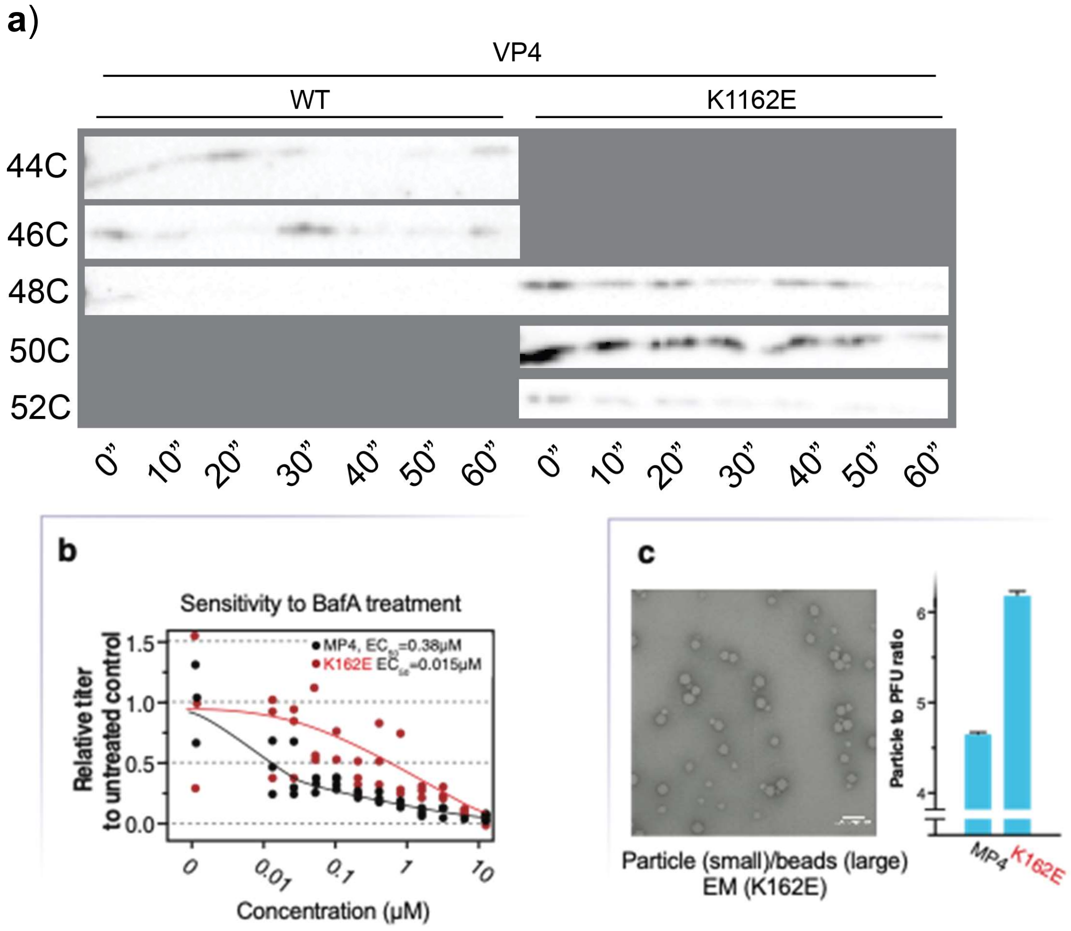

Fig. 2. Particle characteristics. **a**) Examining the presence of VP4 in WT and K162E particles. Purified virus particles were heated at different temperatures, and samples were obtained at 0, 10, 20, 30 , 40, 50 and 60 minutes, digested with trypsin for 30 minutes, and examined for the presence of VP4 by western blotting. While the quality of the Westernblotting is not ideal due to the sammm size and chemical nature of VP4, it is possible to appreciate the presence of VP4 at 50 and 52ºC. In contrast VP4 was undetectable at following 10' incubation at 48ºC. **b**) To determine effective concentration of BafA1 required to inhibit viral entry, we used different concentrations of BfaA1 in RD cells prior to WT and K162E infection. BafA1 was added at least 1 hour prior, and cells washed prior to the addition of virus. Infected cells were allowed to replicate virus until 8 hours post-infection where the number of successful replications was determined by $TCID_{50}$. The EC50 of inactivation was found to be 0.38 M for WT and 0.015 M for K162E, representing approximately a 20-fold increase in inhibition. **c**) To determine if the number of physical particles required for a successful infection was also affected, the specific infectivity viral particles (physical particles to infectious units) was calculated. The concentration of physical particles was measured against a 50-nm latex bead standard of known concentration, and the concentration of infectious particles was measured by plaque assay. The number of physical units required to initiate a

successful infection, in cell culture, was increased about 20-fold by the introduction of K162E.

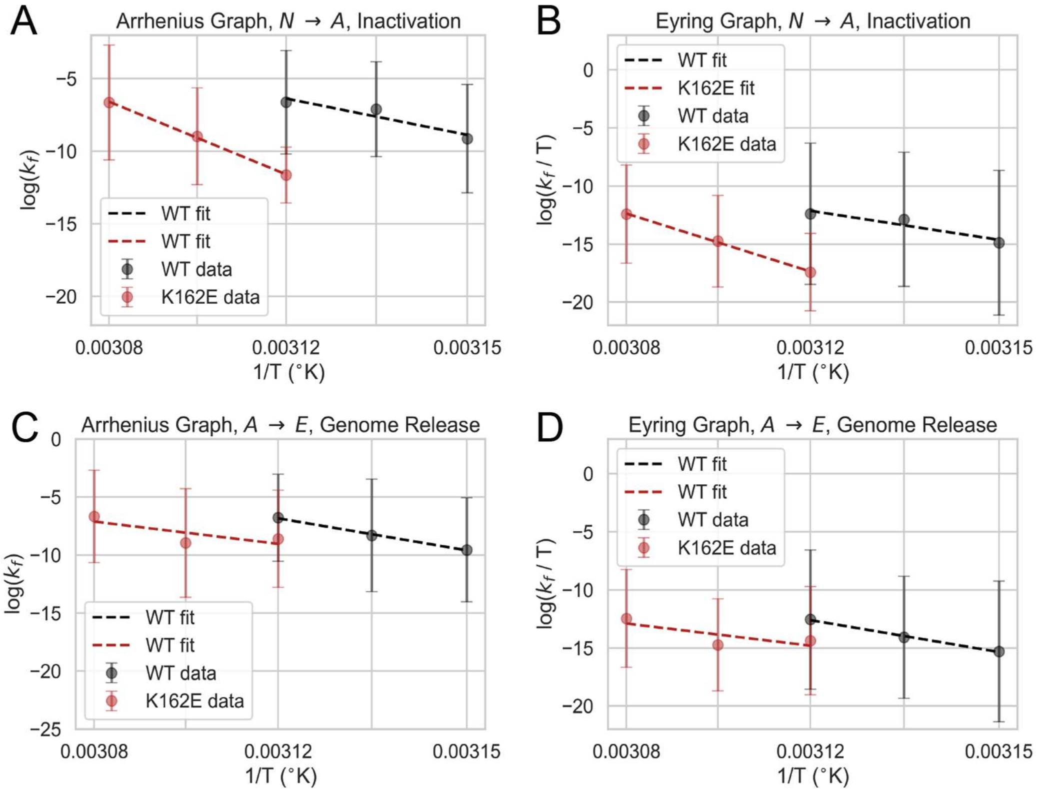

Fig. 3. Arrhenius and Eyring plots of inactivation and uncoating. A) Log-normalized rates of inactivation of WT and K162E from 44° to 52°C. Rates were curve-fit from data in Figs. 3a and 3b using log-normalized least squares. The relationship between log-normalized inactivation rates and the inverse of temperature was fitted by log-normalized least-squares to determine the slope of the curve, yielding the activation energy (E_a). B) Log-normalized rates of inactivation for WT and K162E over the respective temperatures. Rates were determined as mentioned in SM-3A. Intercept of fitted curve was used to determine the enthalpy ($\boldsymbol{\Delta}H^{\dagger}$) from the slope of the curve and the entropy ($\boldsymbol{\Delta}S^{\dagger}$) from the intercept of the curve. C) Log-normalized rates of genome release of WT and K162E from 44° to 52°C. Rates and curve-fitting was performed as described in SM-3A. D) Log-normalized rates of genome release for WT and K162E over the respective temperatures. Rates and curve-fitting was performed as described in SM-3.

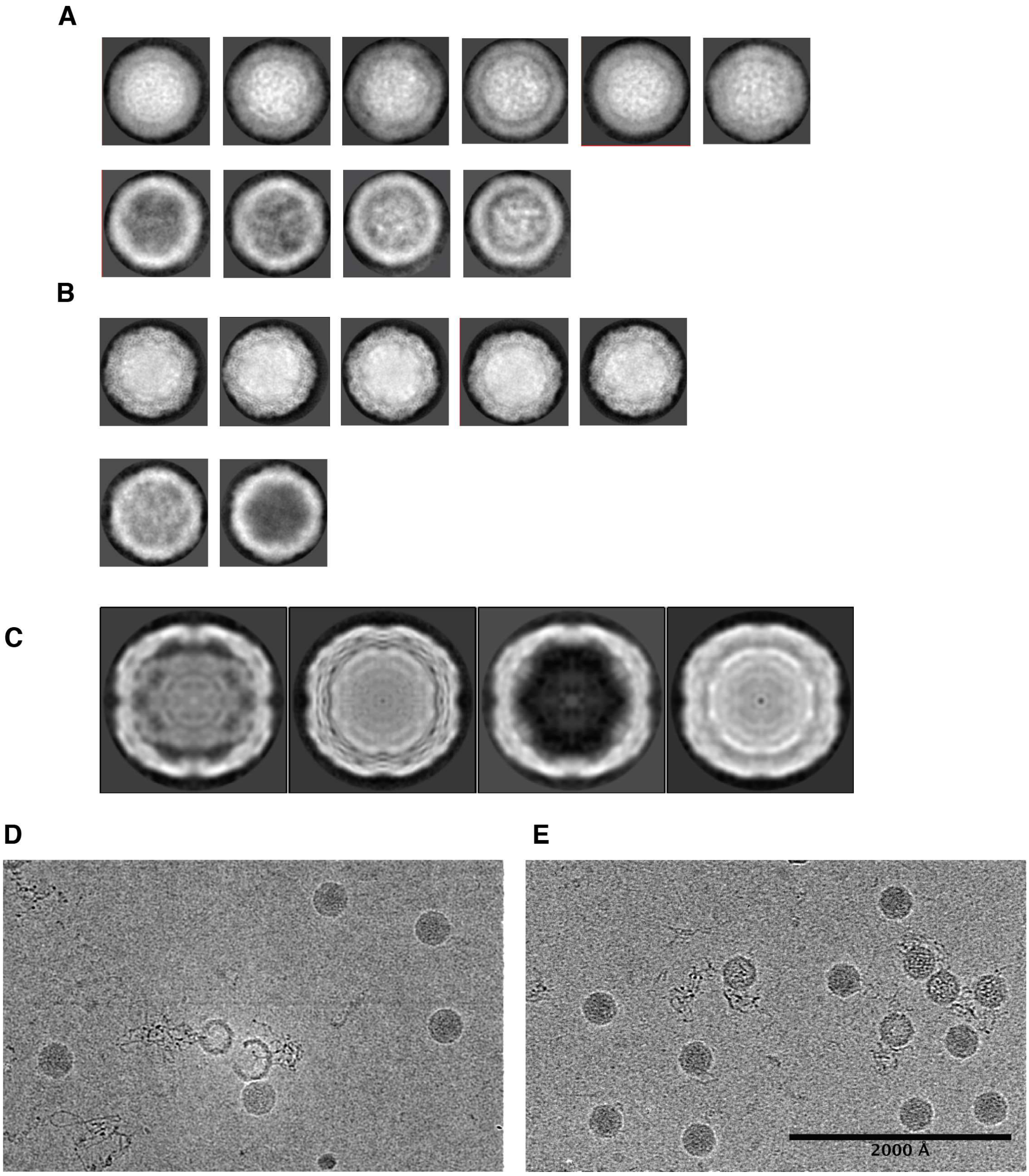

Fig. 4. Images of heated WT and K162E particles. A) 2D class averages of heated WT particles, either used for A-particle (top row) or empty particle (bottom row) reconstruction. B) 2D class averages of heated K162E particles, either used for pre-A, A-particle, or post-A (top row) or empty particle (bottom row) reconstructions. C) Projections of 3D class averages of heated K162E particles. From left to right, projections are of empty, A-particle, post-A, and pre-A states. D) Sample micrograph of heated WT particles. E) Sample micrograph of heated K162E particles.

	WT-native	WT-A-particle	WT-empty	K162E-native	K162E-A_1-particle	K162E-A_2-particle	K162E-A_3-particle	K162E-empty
# particles	20699	1316	848	12966	2094	604	246	162
Map resolution (A)	3.3	8	14	4.1	3.1	7.4	5.9	7.1
Detector pixel size (A)	0.72	0.72	0.72	0.603	0.72	0.72	0.72	0.7
Nominal magnification	28000	28000	28000	34000	28000	28000	28000	28000
Electron exposure (e^-/A^2)	62	62	62	68	62	62	62	62
Voltage (kV)	200	200	200	200	200	200	200	200
**Atomic Model**								
Cross-Correlation	0.85	0.62		0.74	0.83	0.64	0.71	0.61
MolProbity Score	1.44	2.01		2.99	1.51	2.19	1.73	2.46
Clash Score	6.43	11.06		10.93	4.49	10.39	7.70	15.57
Poor Rotamers	0.14	0.17		8.41	0	0	0	8.35
Favored Ramachandran	97.6	92.95		79.86	96.04	91.73	95.5	96.55
Allowed Ramachandran	2.4	6.9		19.42	3.96	8.27	4.5	3.45
Disallowed Ramachandran	0	0.15		0.72	0	0	0	0

Fig. 5. Summary statistics of cryo-EM and atomic model building

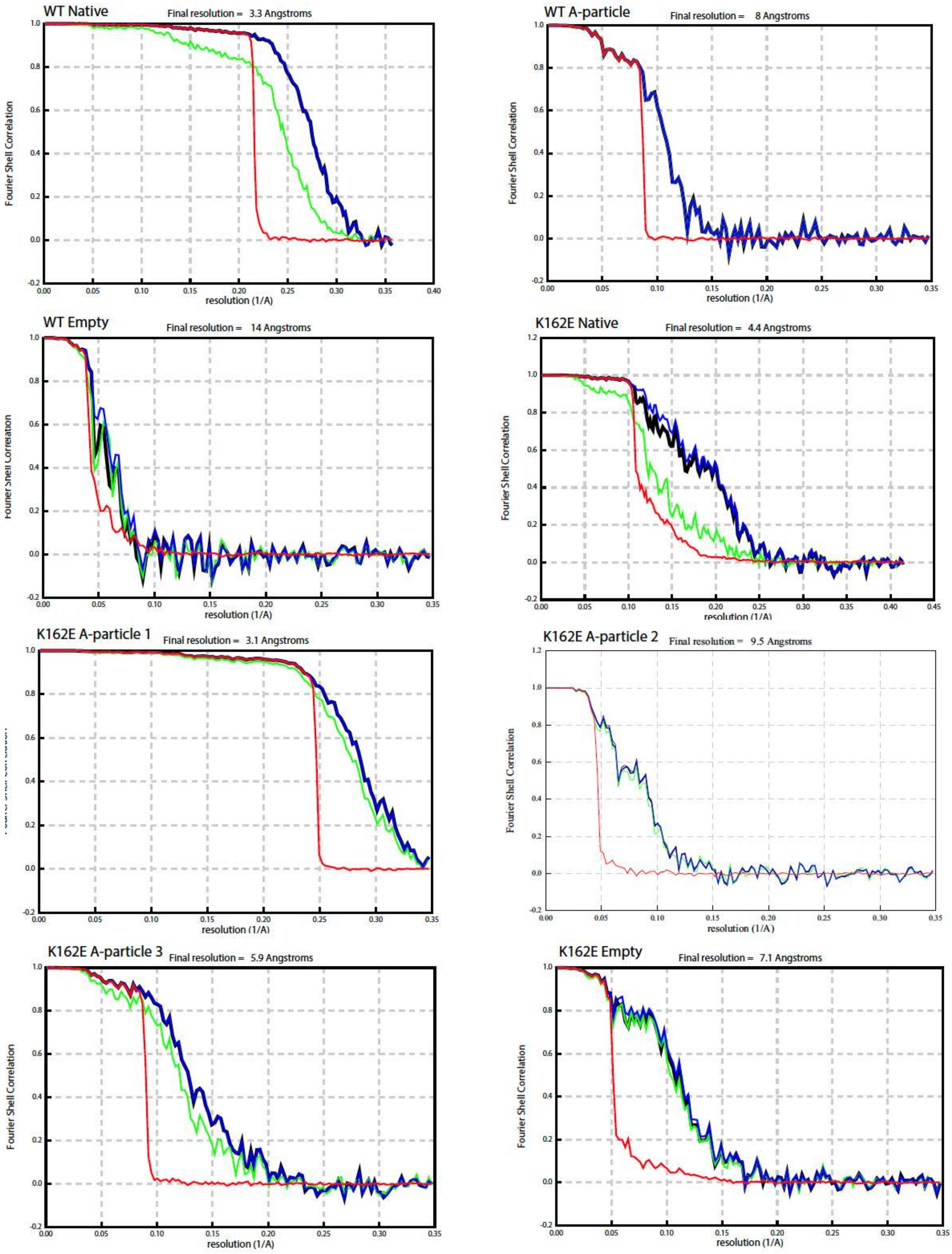
WT Native
Final resolution = 3.3 Angstroms
WT A-particle
Final resolution = 8 Angstroms
WT Empty
Final resolution = 14 Angstroms
K162E Native
Final resolution = 4.4 Angstroms
K162E A-particle 1
Final resolution = 3.1 Angstroms
K162E A-particle 2
Final resolution = 9.5 Angstroms
K162E A-particle 3
Final resolution = 5.9 Angstroms
K162E Empty
Final resolution = 7.1 Angstroms
Fourier Shell Correlation
resolution (1/A)

Fig. 6. Fourier shell correlations (FSCs) of structures determined. Final resolution calculated by gold-standard 0.143 cutoff of masked maps (blue). Also shown are FSC corrected (black), FSC unmasked (green), and FSC phase-randomized masked (red).

MD simulation results

From molecular dynamics simulations, it is possible to track each single atom of a system, which is useful to provide insights into the microscopic features of a system, in our case on EV-A71 WT and K162E respectively (Fig. 7). We computed the root mean-squared deviations (RMSDs) (Fig. 8-13) which is a measure of the average displacement of a protein from its initial configuration. On average we observe that at both 30 and 52 C, the local dynamics of the K162E is greater than that of the WT, indicating the higher mobility of the mutant, which enables to dissipate the thermal energy allowing the mutant to be still active and functioning at high temperatures.

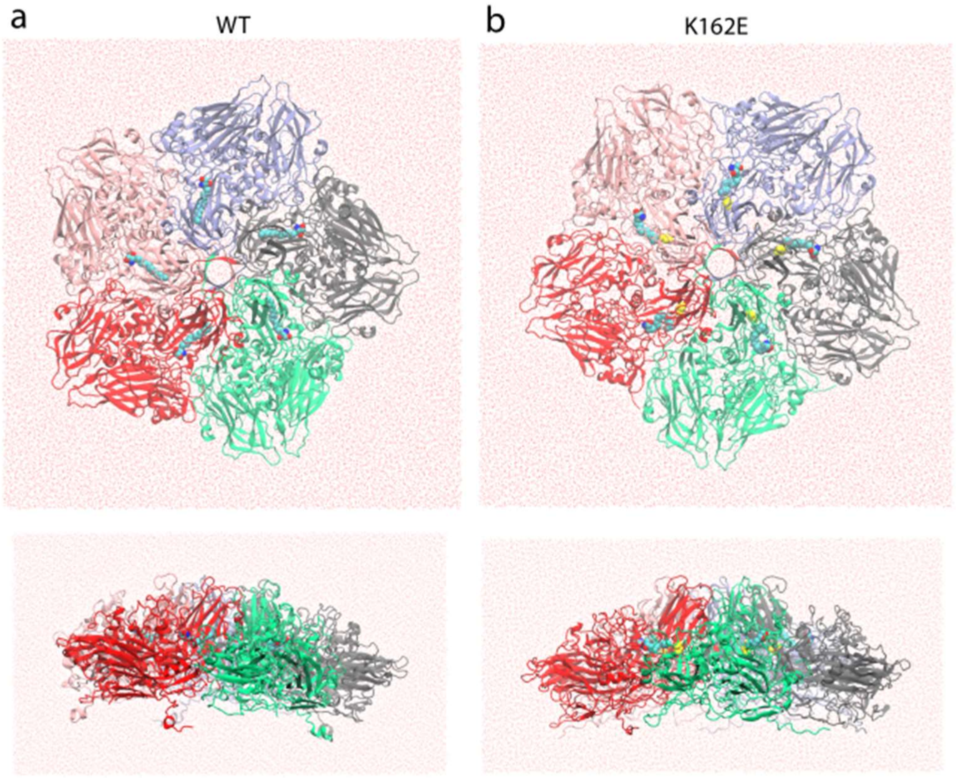

Fig. 7. Top and side views of the initial structure of water-simulated box of the WT (a) and K162E (b) capsomeres forming the pentamer. The five capsomeres formed by VP1, VP2, VP3, and VP4 capsid proteins are colored differently. In both systems, sphingosine is represented in van Der Waals format (hydrogen atoms are hidden for clarity) and in the K162E system the VP1 residue 162 is colored yellow and represented in van Der Waals format (hydrogen atoms are hidden for clarity).

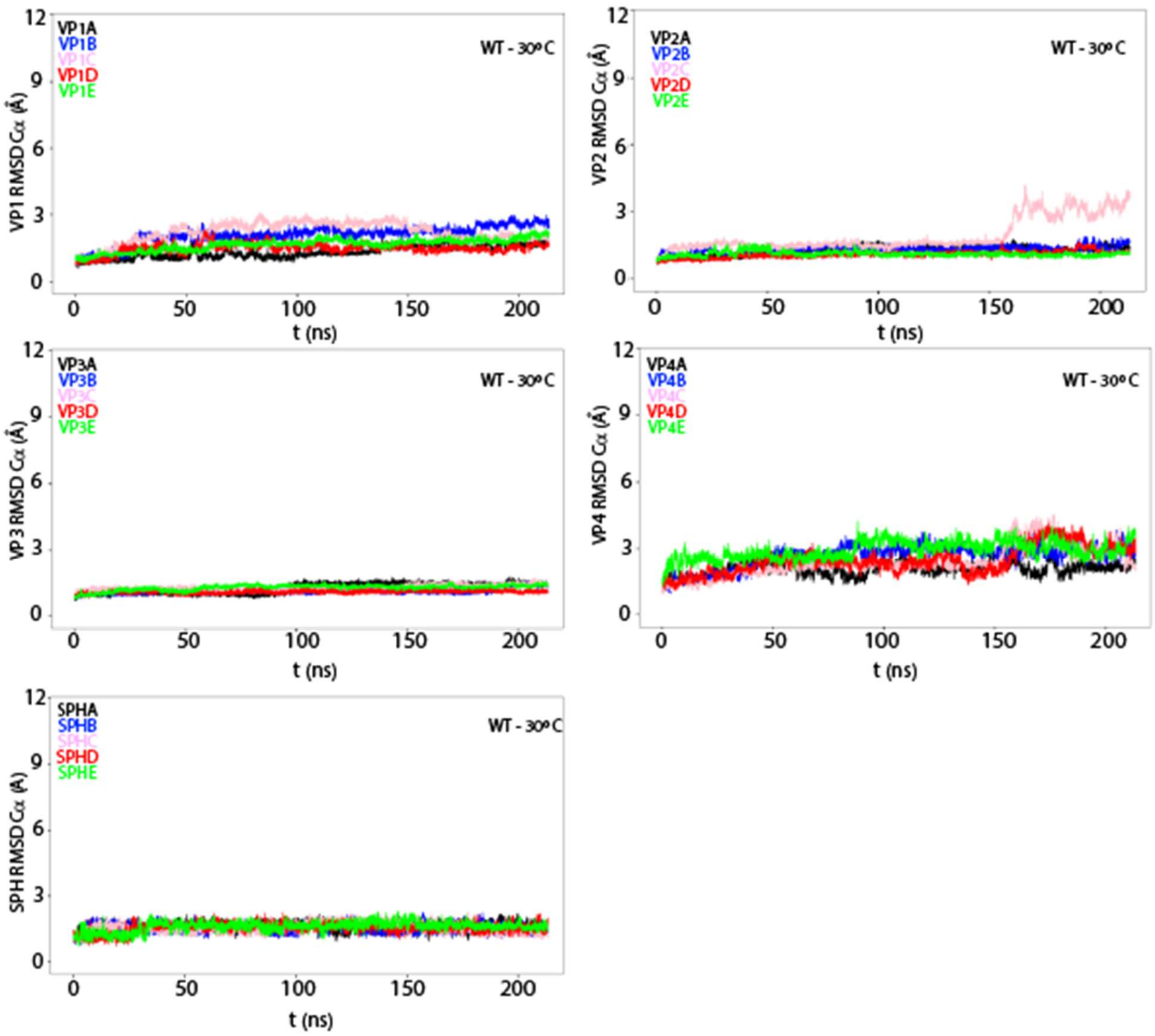

Fig. 8. Time evolution of the root mean-squared deviations (RMSD) of the VP1, VP2, VP3, and VP4 capsid proteins and of the sphingosine pocket factor for NPT simulations of the EV-A71 WT carried out at 30ºC. The panels show the time evolution of the RMSD of all the capsid proteins and sphingosine of the five protomers colored differently (protomer A: black; B: blue; C: pink; D: red; E: green).

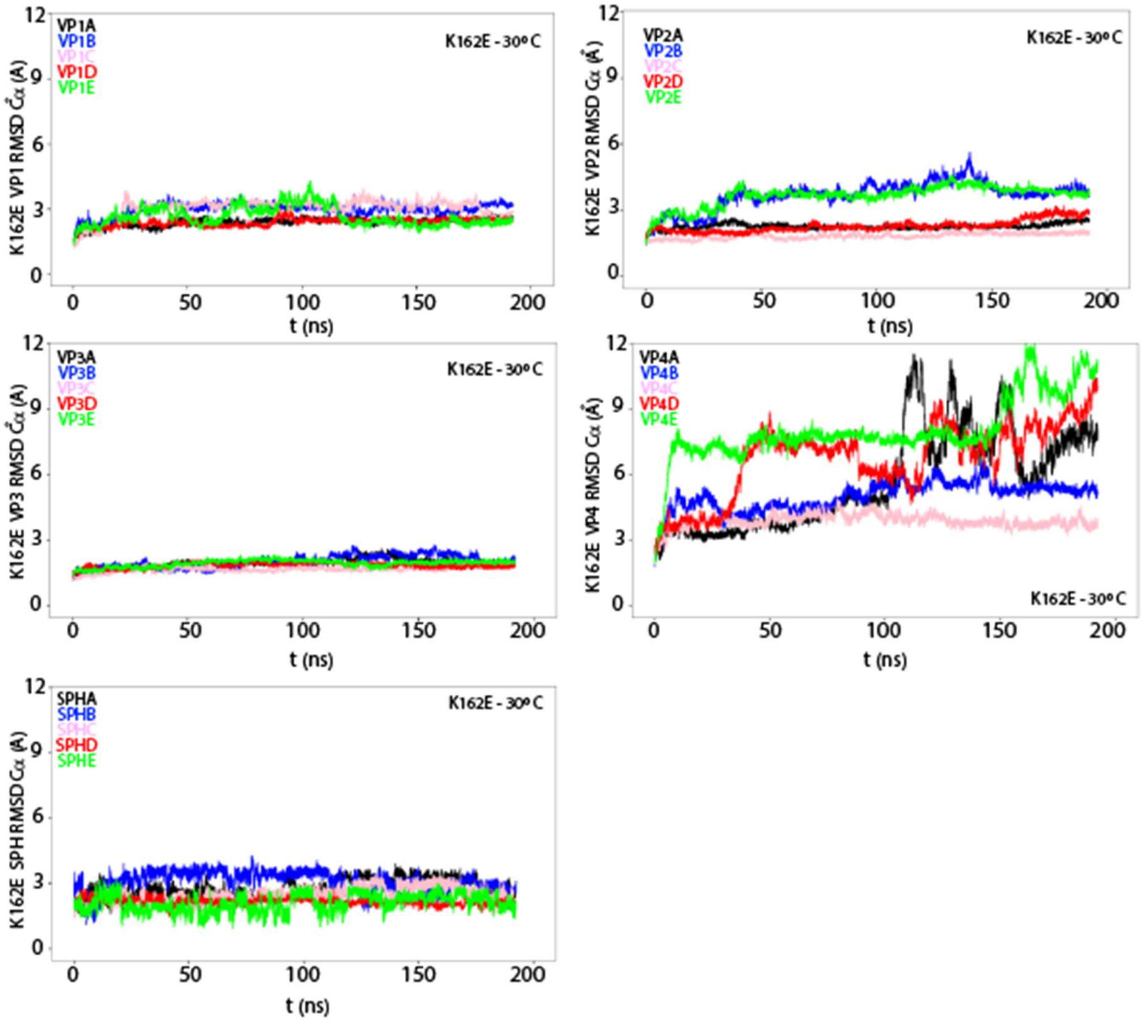

Fig. 9. Time evolution of the root mean-squared deviations (RMSD) of the VP1, VP2, VP3, and VP4 capsid proteins and of the sphingosine pocket factor for NPT simulations of the EV-A71 K162E mutant carried out at 30ºC. The panels show the time evolution of the RMSD of all the capsid proteins and sphingosine of the five protomers colored differently (protomer A: black; B: blue; C: pink; D: red; E: green)

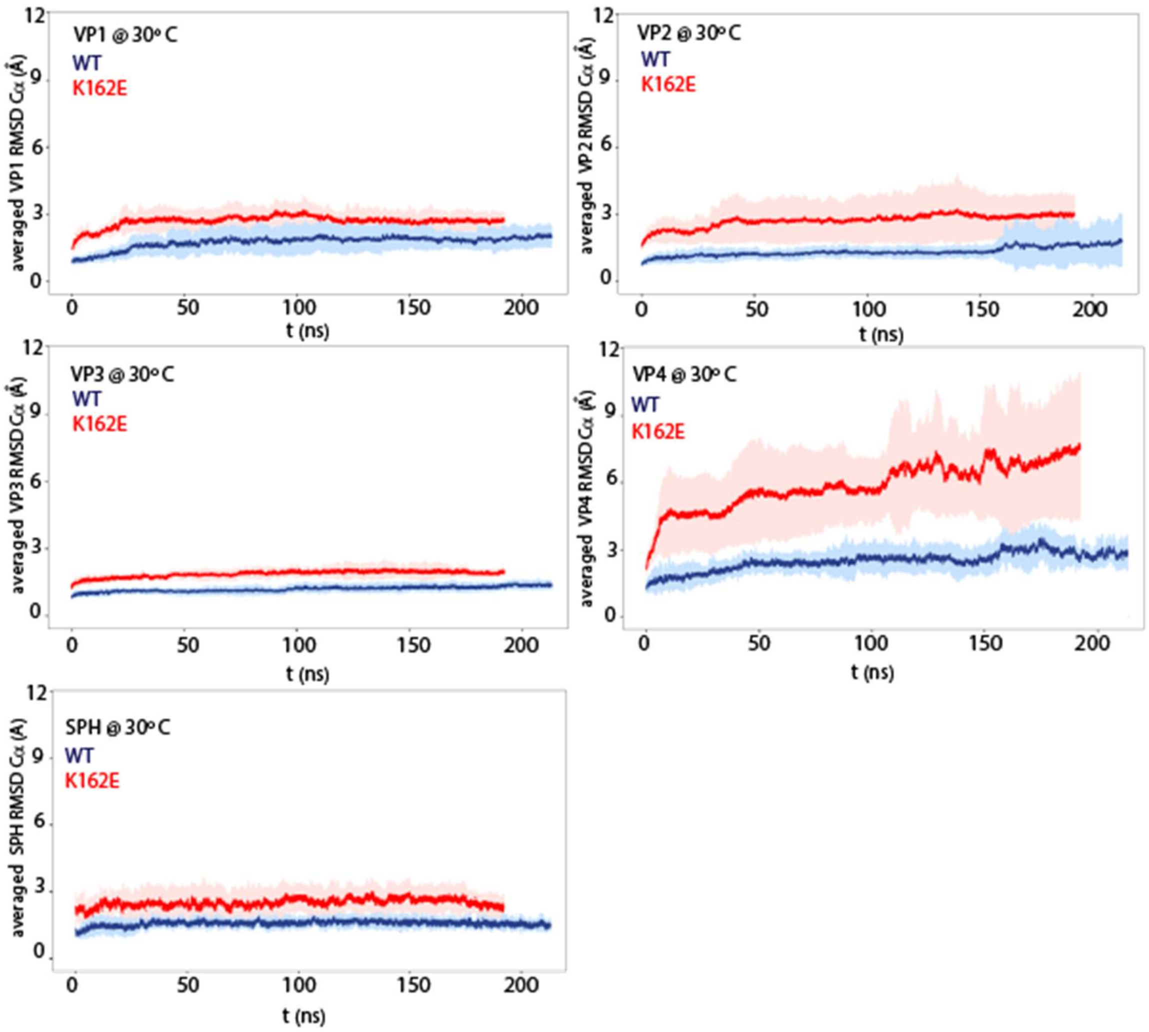

Fig. 10. Time evolution of the mean value root mean-squared deviations (RMSD) at 30ºC calculated by averaging the RMSD value at each time point of the simulations of VP1, VP2, VP3, VP4 capsid protein, and of the sphingosine of each protomer. The error bars represent the standard deviation of the mean.

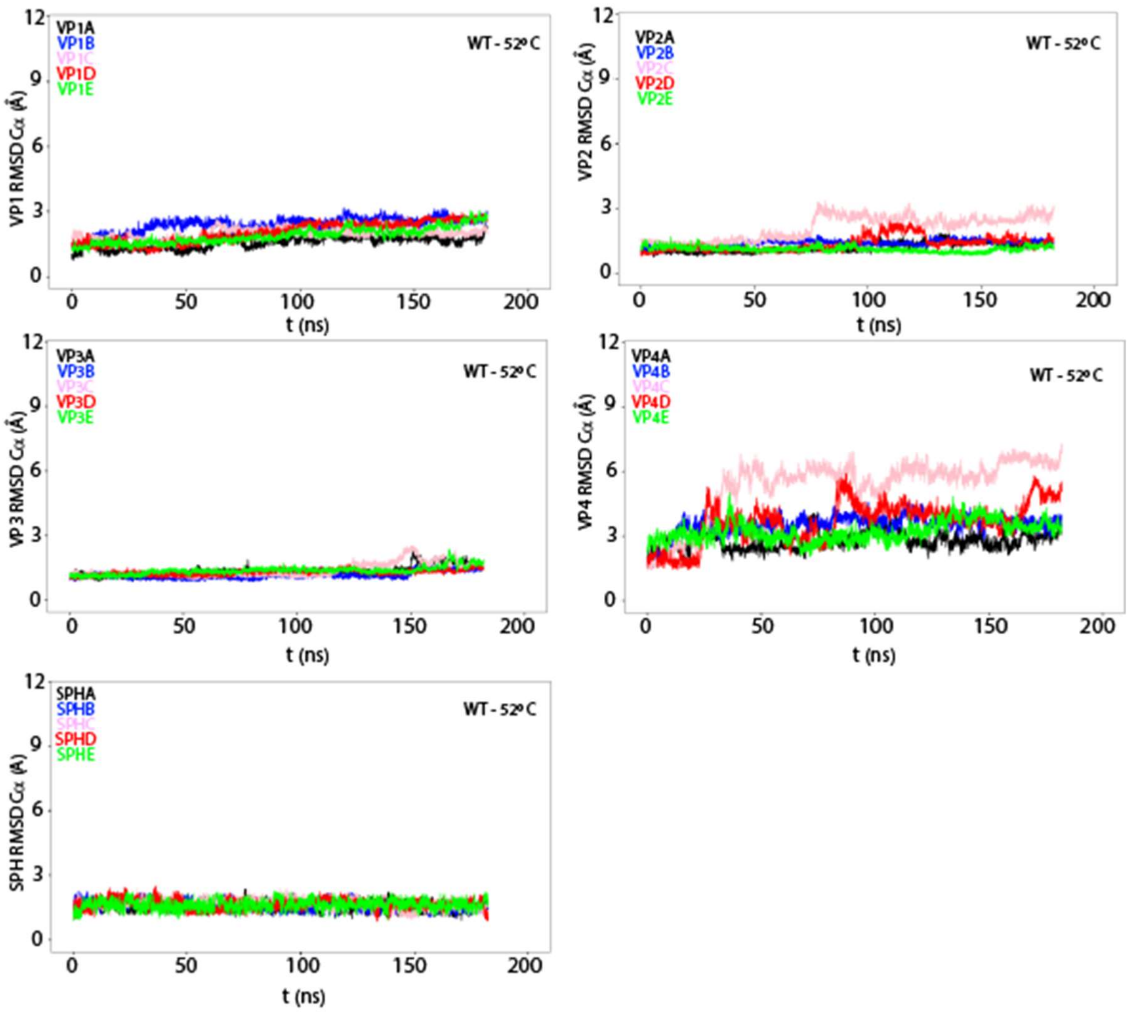

Fig. 11. Time evolution of the root mean-squared deviations (RMSD) of the VP1, VP2, VP3, and VP4 capsid proteins and of the sphingosine pocket factor for NPT simulations of the EV-A71 WT carried out at 52ºC. The panels show the time evolution of the RMSD of all the capsid proteins and sphingosine of the five protomers colored differently (protomer A: black; B: blue; C: pink; D: red; E: green)

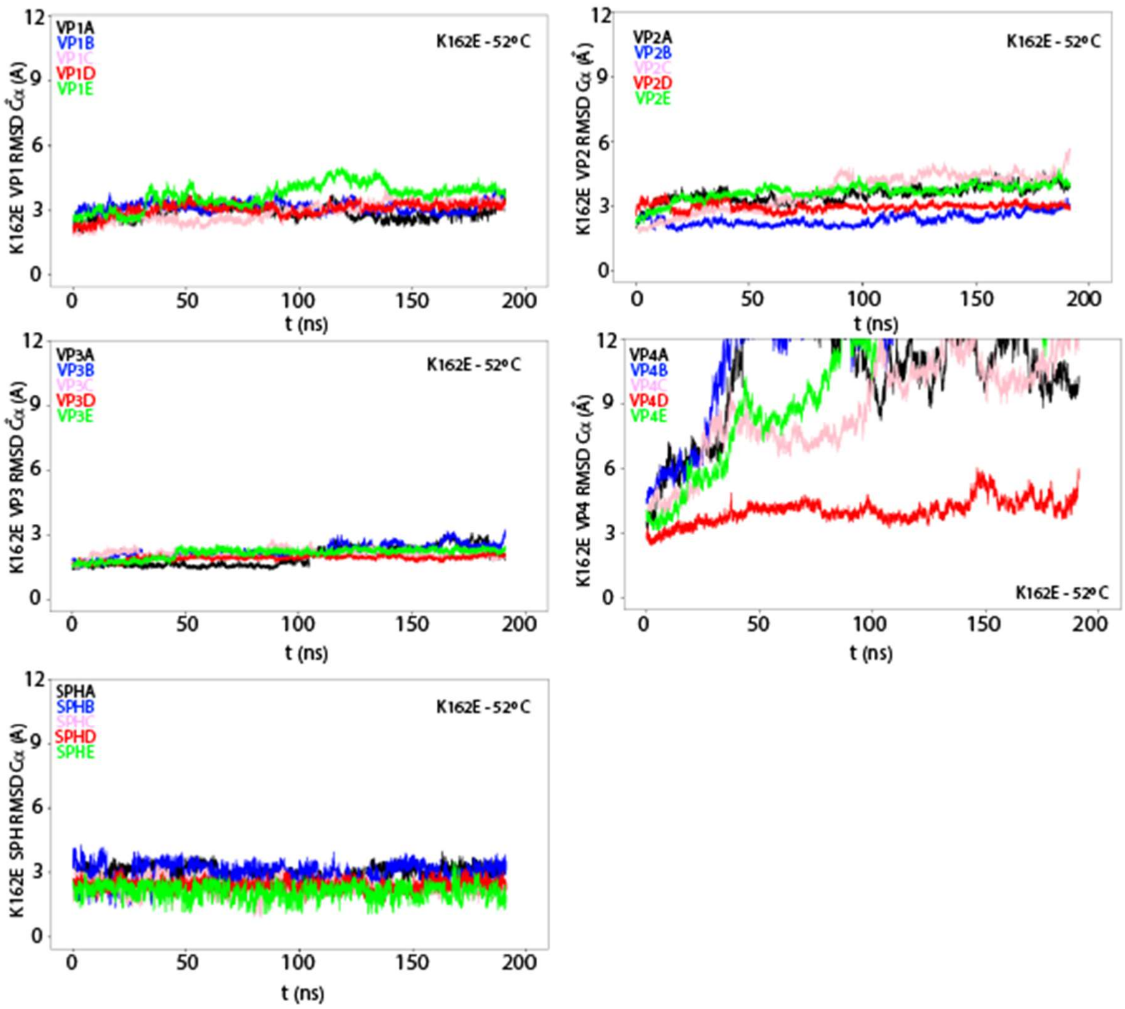

Fig. 12. Time evolution of the root mean-squared deviations (RMSD) of the VP1, VP2, VP3, and VP4 capsid proteins and of the sphingosine pocket factor for NPT simulations of the EV-A71 K162E mutant carried out at 52ºC. The panels show the time evolution of the RMSD of all the capsid proteins and sphingosine of the five protomers colored differently (protomer A: black; B: blue; C: pink; D: red; E: green)

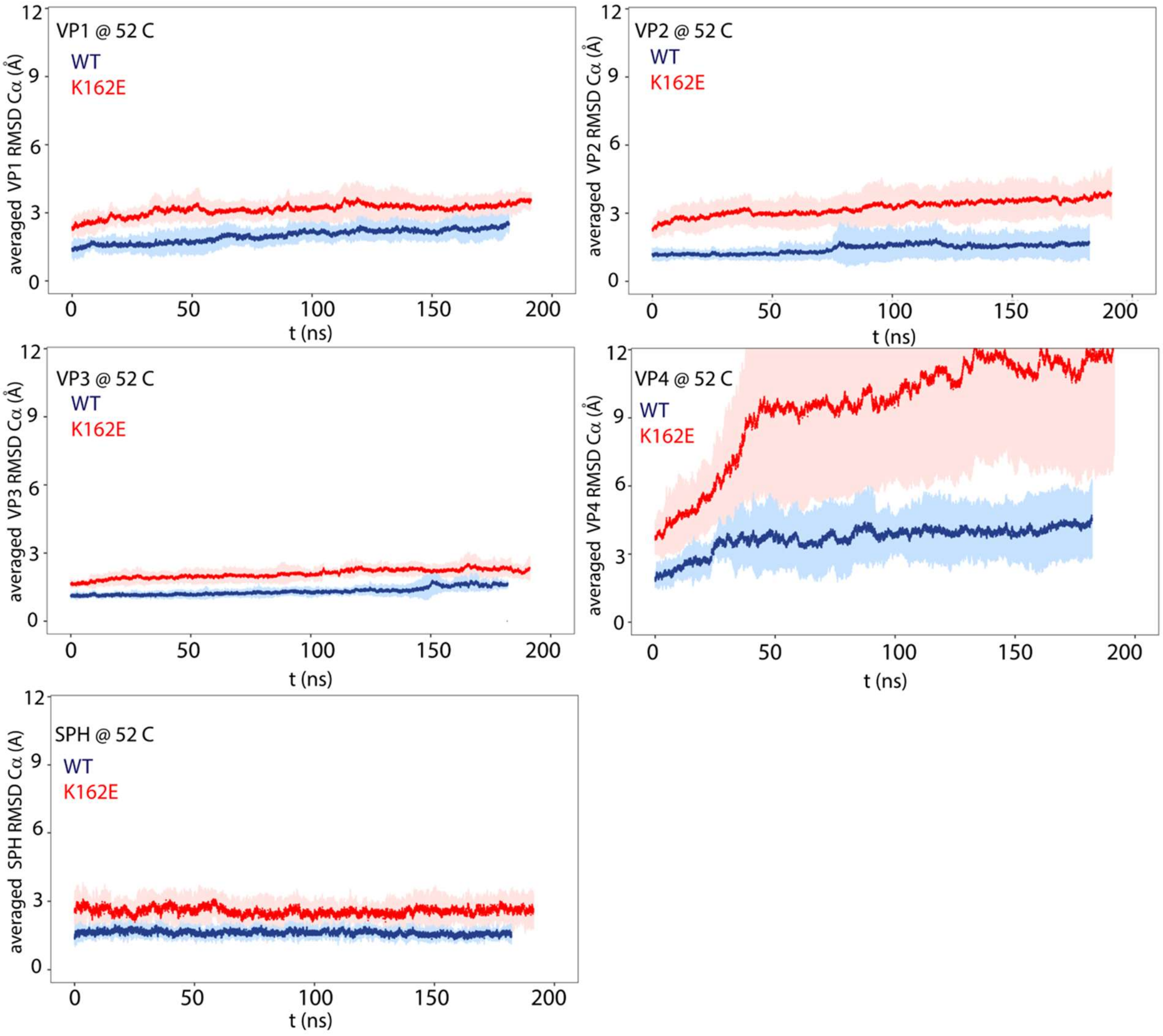

Fig. 13. Time evolution of the mean value root mean-squared deviations (RMSD) at 52ºC calculated by averaging the RMSD value at each time point of the simulations of VP1, VP2, VP3, VP4 capsid protein, and of the sphingosine of each protomer. The error bars represent the standard deviation of the mean.

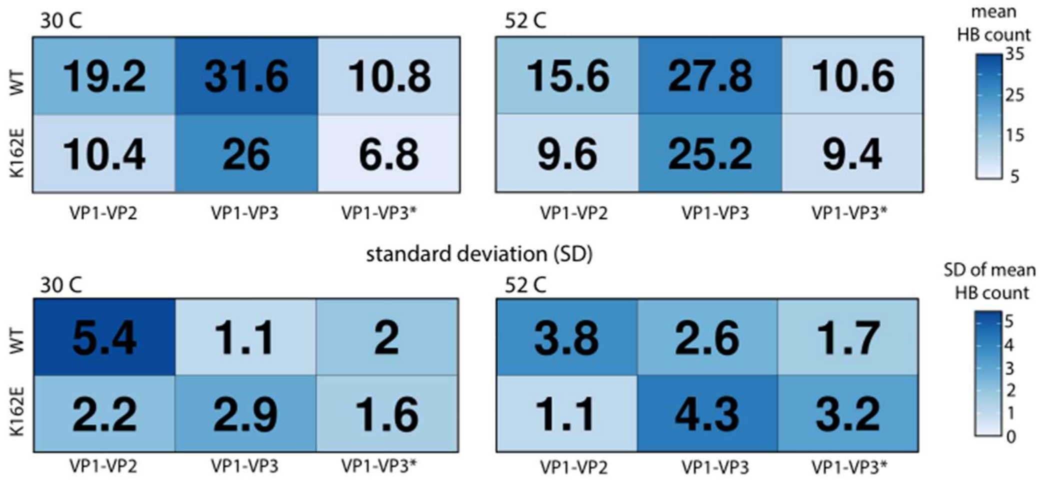

Fig. 14. Top panels: Mean of the number of the hydrogen bonds between VP1-VP2 and VP1-VP3 capsid proteins belonging to the same protomer and between VP1-VP3 capsid proteins belonging to adjacent protomers (VP1-VP3*) calculated for the WT and K162E mutant at 30ºC (left panel) and 52ºC (right panel). The calculation has been performed during the last 5 ns of the simulations. Bottom panels: the standard deviation (SD) of the mean of the hydrogen bond (HB) count is represented. The standard deviation is evaluated by considering the five protomers forming the pentamer.

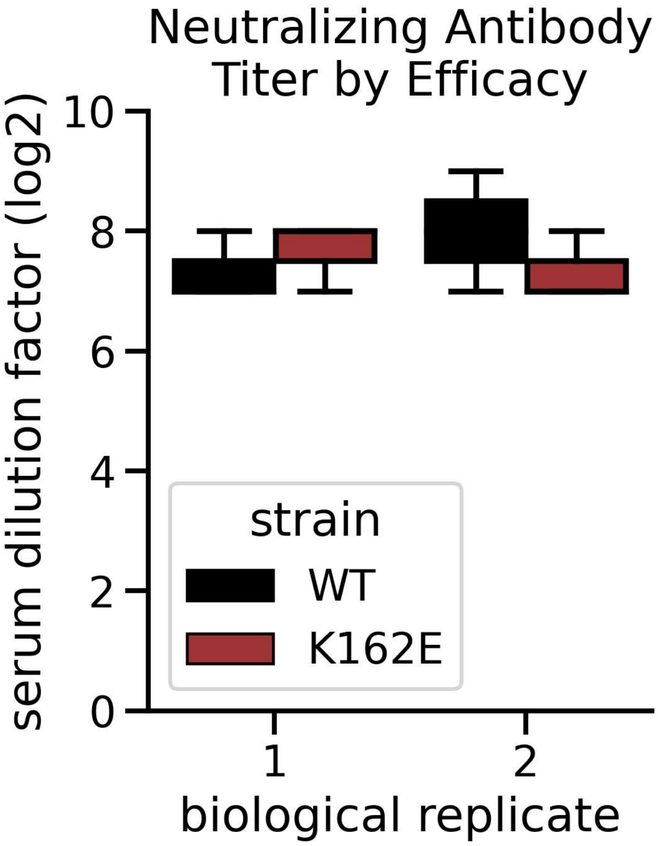

Fig. 15. WT and E162K are equally neutralized by polyclonal serum. Either WT or K162E was incubated with different dilutions of serum from a mouse infected with EV-A71. Maximum dilution factor at which neutralization was observed is indicated. Serum was collected from two independent mice and used in biological duplicate, reported dilutions were performed in technical triplicate.

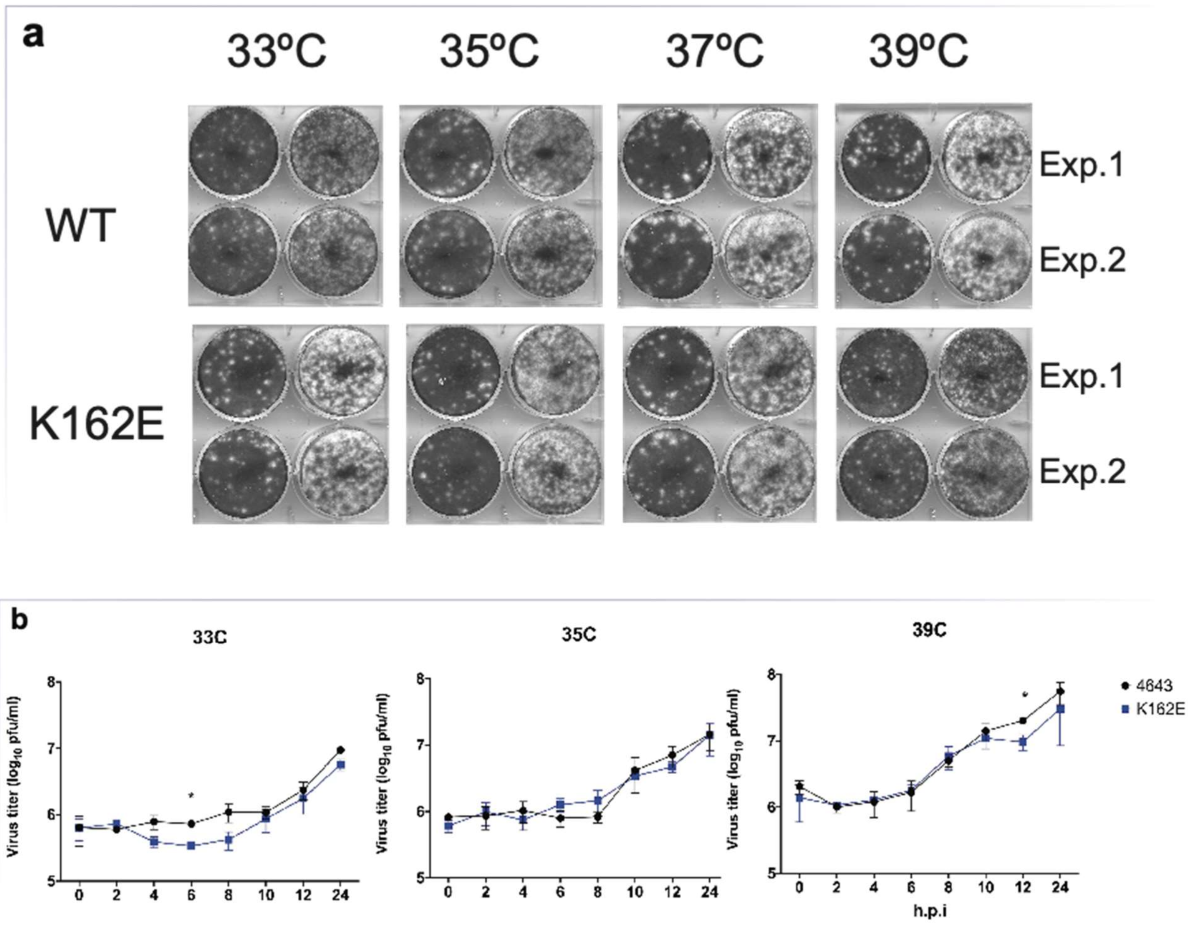

Fig. 16. Plaque morphology under various temperatures. (a) RD cells were infected with the WT (strain 4643) or 4643-K162E at m.o.i. of 5 and 0.5 and cultured at 33℃, 35℃, 37℃, and 39℃ to determine plaque morphology under different temperatures. The plaques shown were from two infections. (b) **Virus replication under various temperatures.** RD cells were infected with the WT (strain 4643) or 4643-K162E viruses at an m.o.i. of 10 and cultured at 33℃, 35℃, 37℃, and 39℃. Viruses were collected at a two-hour interval until 12 hours post-infection and at 24 hours for a standard TCID50 assay to determine virus titers. The experiment was performed in triplicate, and the data was shown as Mean with S.D. Statistical analysis was performed using a multiple T-test.

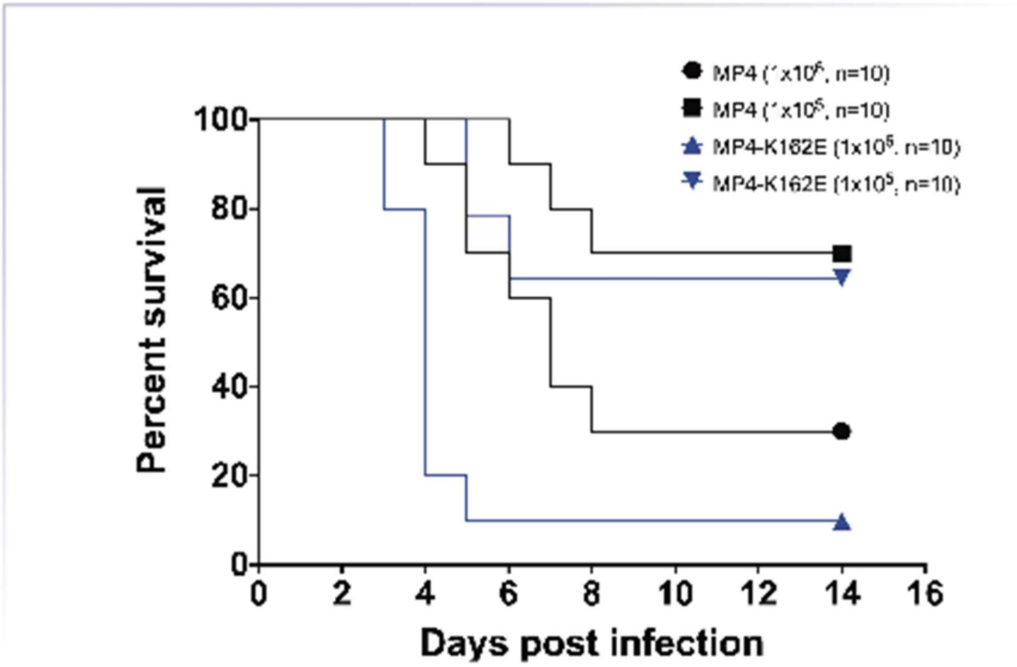

Fig. 17. Mouse Virulence test. Eight-week-old hSCARB2 mice were intraperitoneally inoculated with 1x10^5-1x10^6 TCID50 of the WT (strain MP4) or MP4-K162E virus and monitored survival for 14 days. Mice that reached the humane endpoint with paralysis observed on both posterior limbs were euthanized.

K162E-native

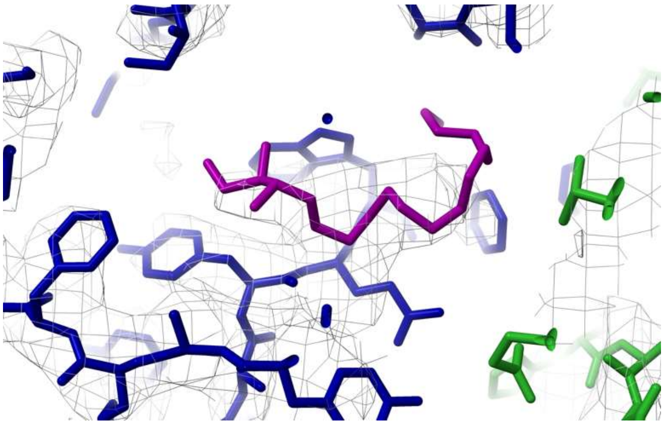

K162E-A1

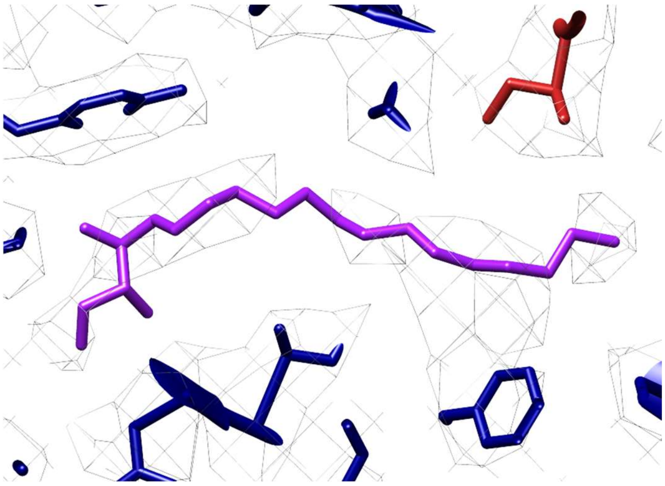

WT-native

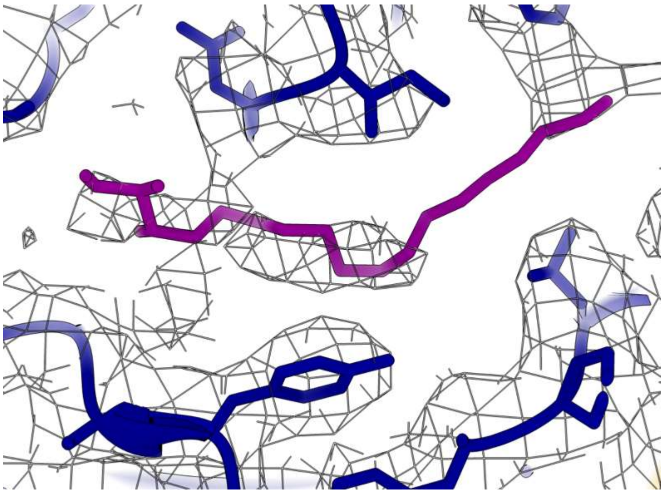

WT-A particle

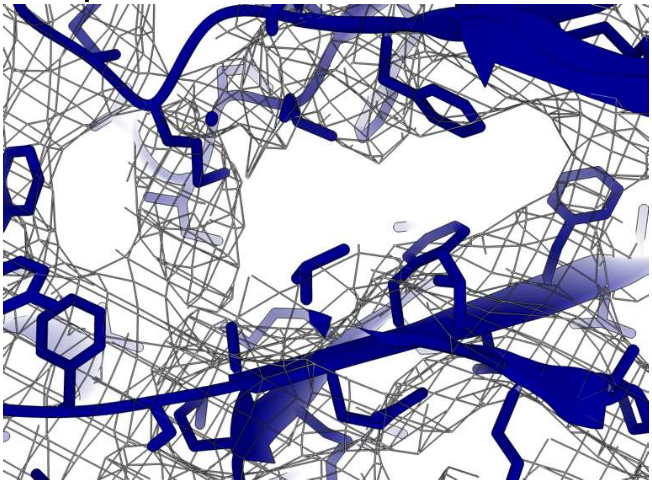

6UH1

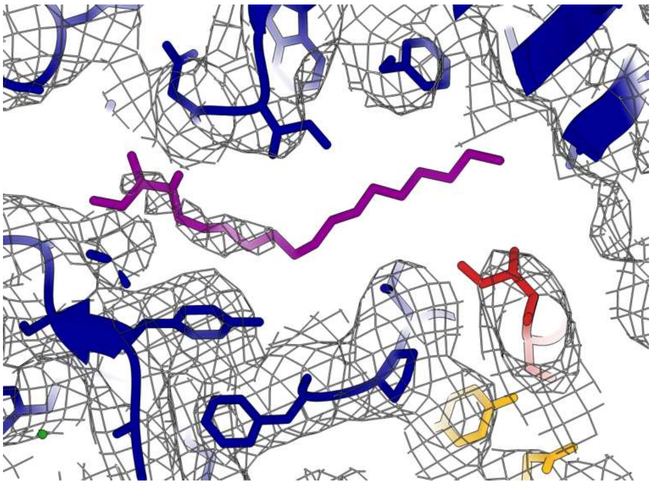

Fig. 18. Modeling the structure and position of pocket factor. We were able to identify Coulomb-density map corresponding to the pocket factor during model building. This density was present in K162E native and A1 particle but the absence of the map density corresponding to the K162E-A2 model. Above are the fitted pocket factors (purple) in the Coulomb-density mesh, surrounded by models of VP1 (blue), VP2 (green), and VP3 (red). While the density for the K162E-native structure is poor, the Coulomb density of the region of the pocket factor cannot be attributed to nearby sidechains. As the pocket factor is a dynamic ligand that leaves the interior of VP1 during the uncoating process, low resolution of the pocket factor is usually observed in enterovirus capsids, as compared with the EM map and model of EV71 structure 6UH1. Thus, similar of Coulomb densities are found in the native WT and K162E structures, as well as the A1 state of K162E, but not in the A-particle state of the WT and K162e A2.

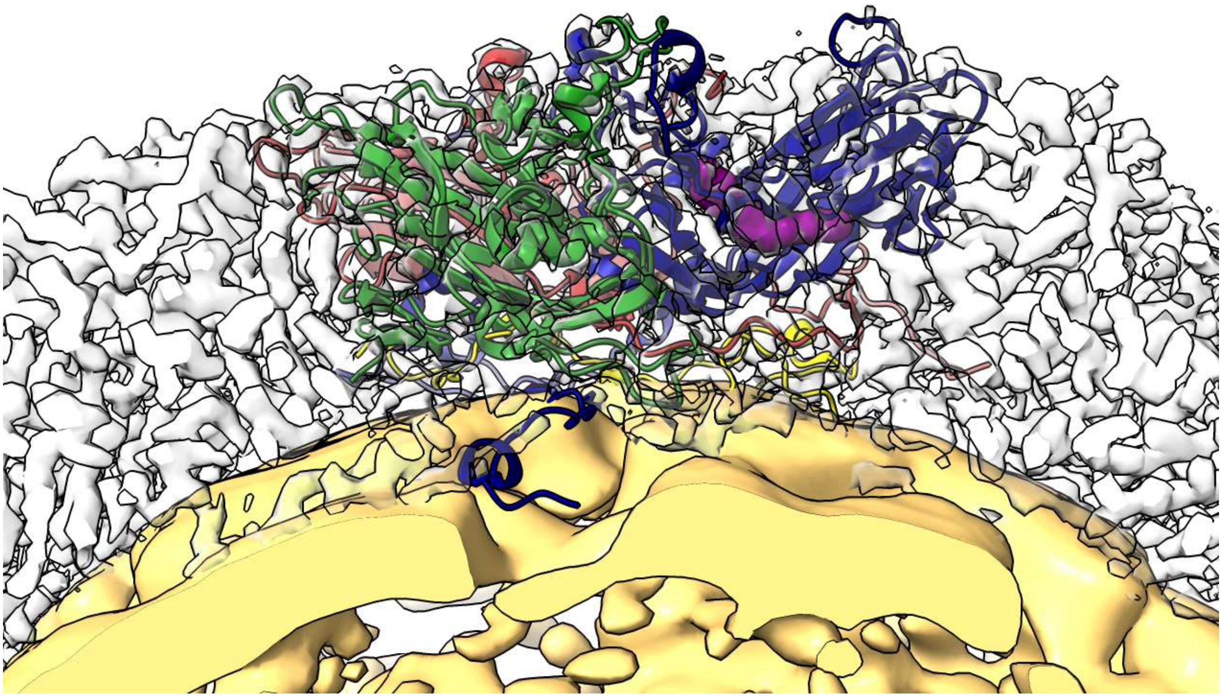

WT-native

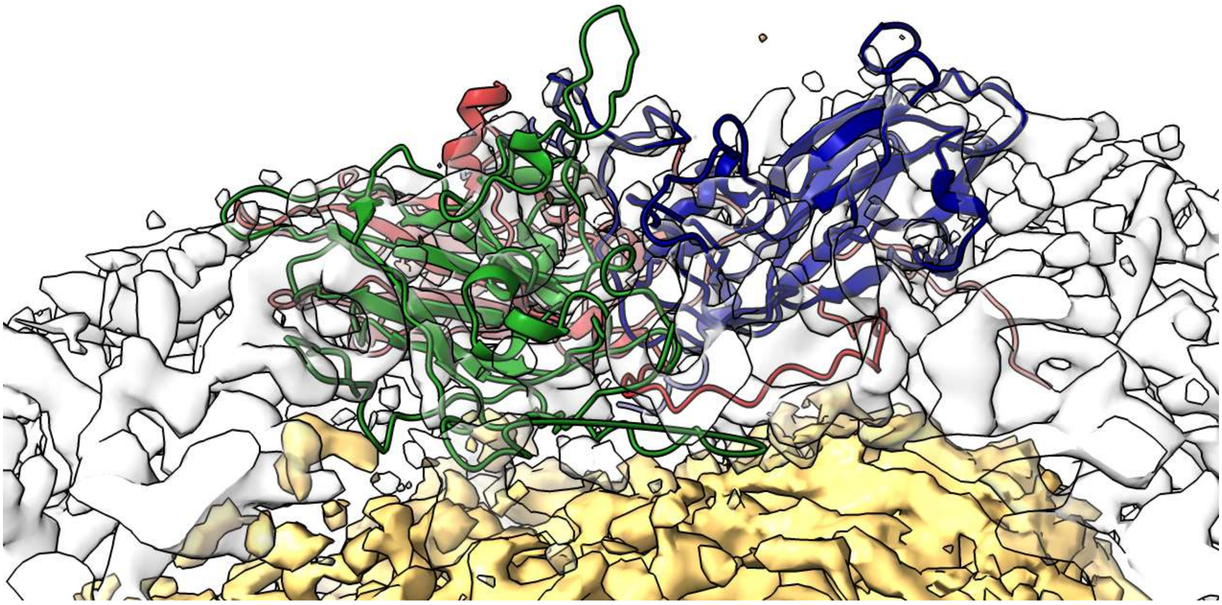

WT-A particle

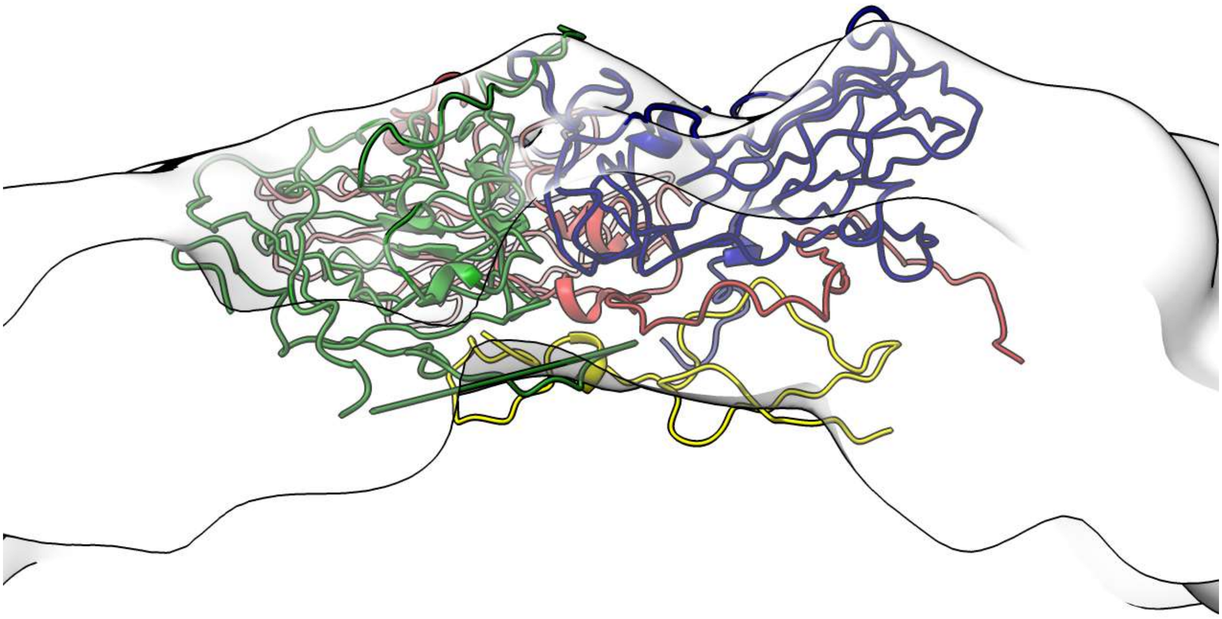

WT-empty

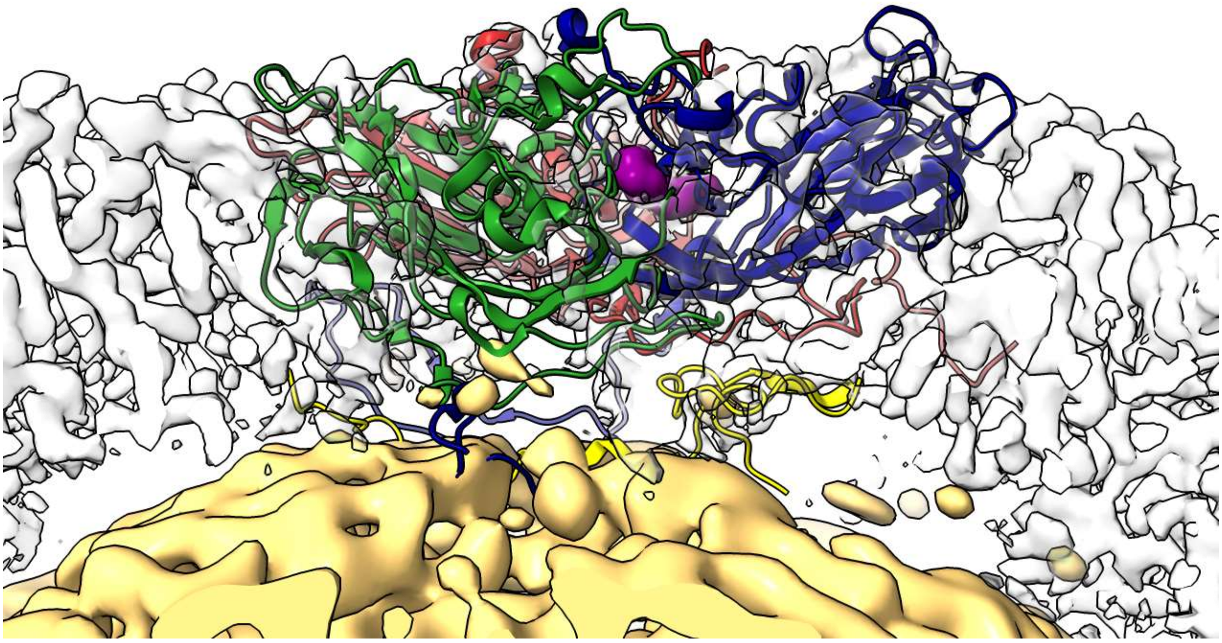

K162E native

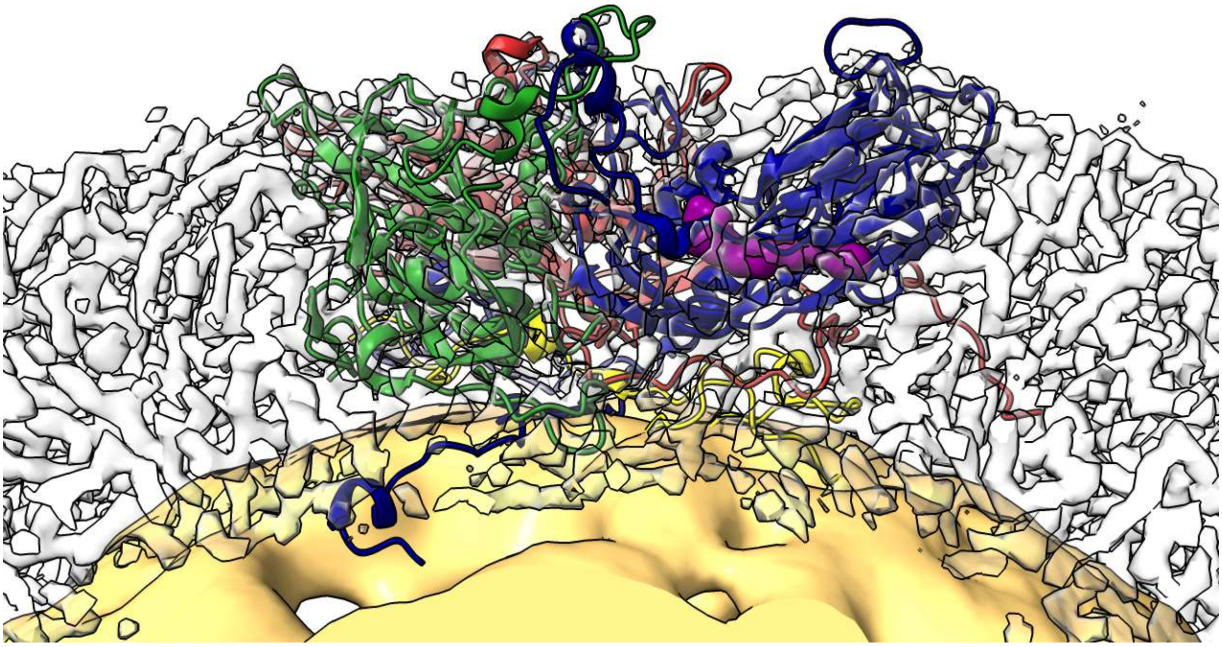

K162E A1 particle

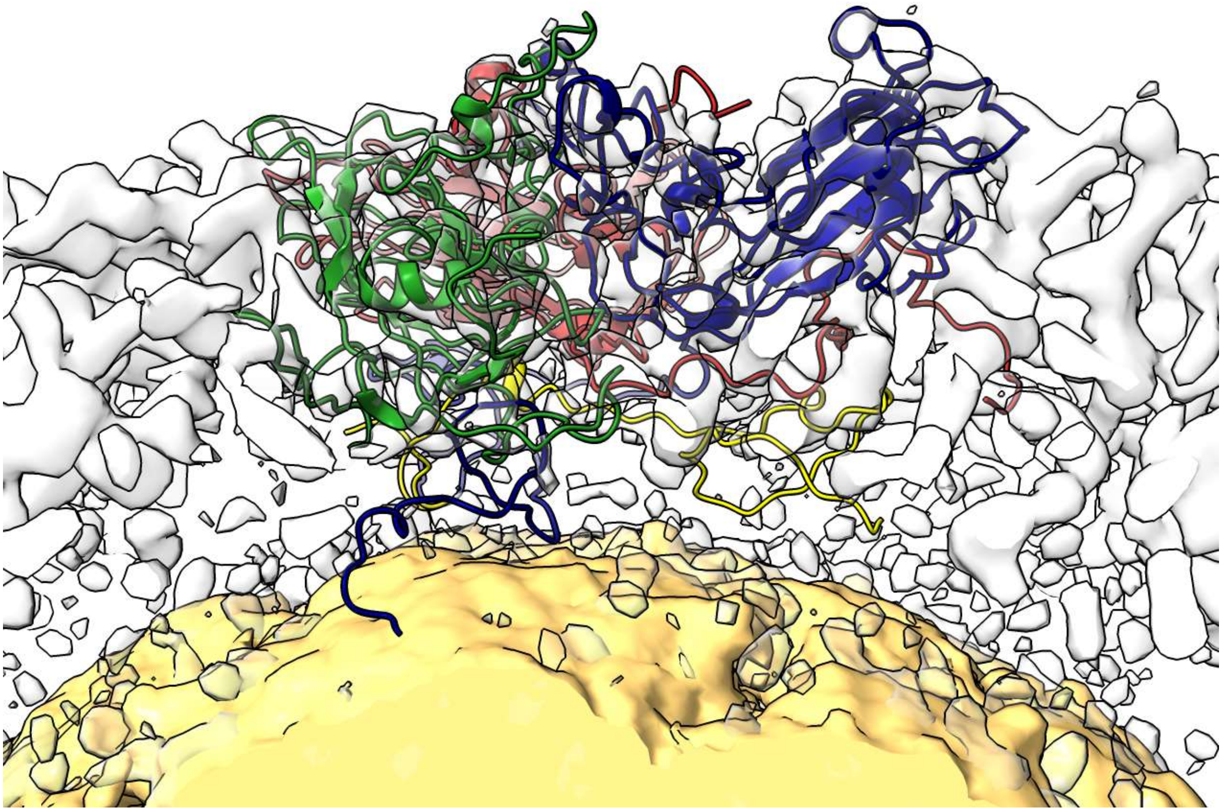

K162E A2 particle

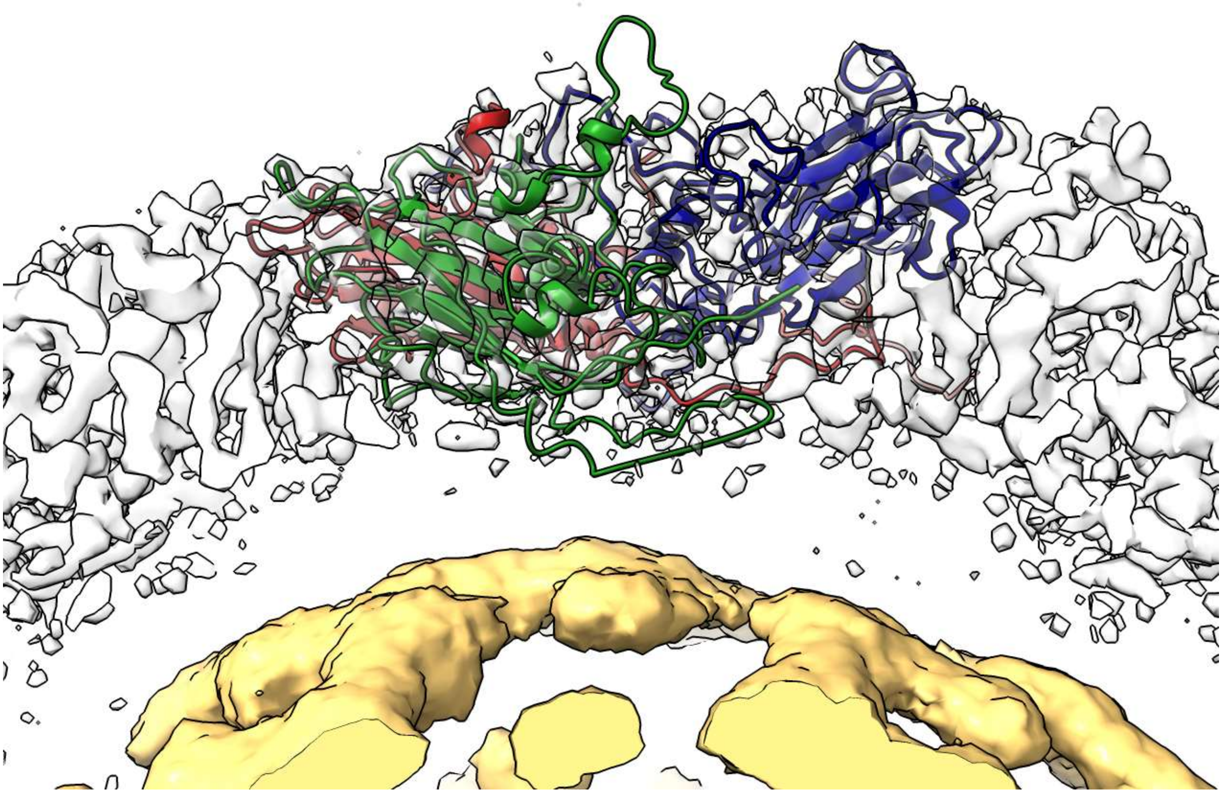

K162E A3 particle

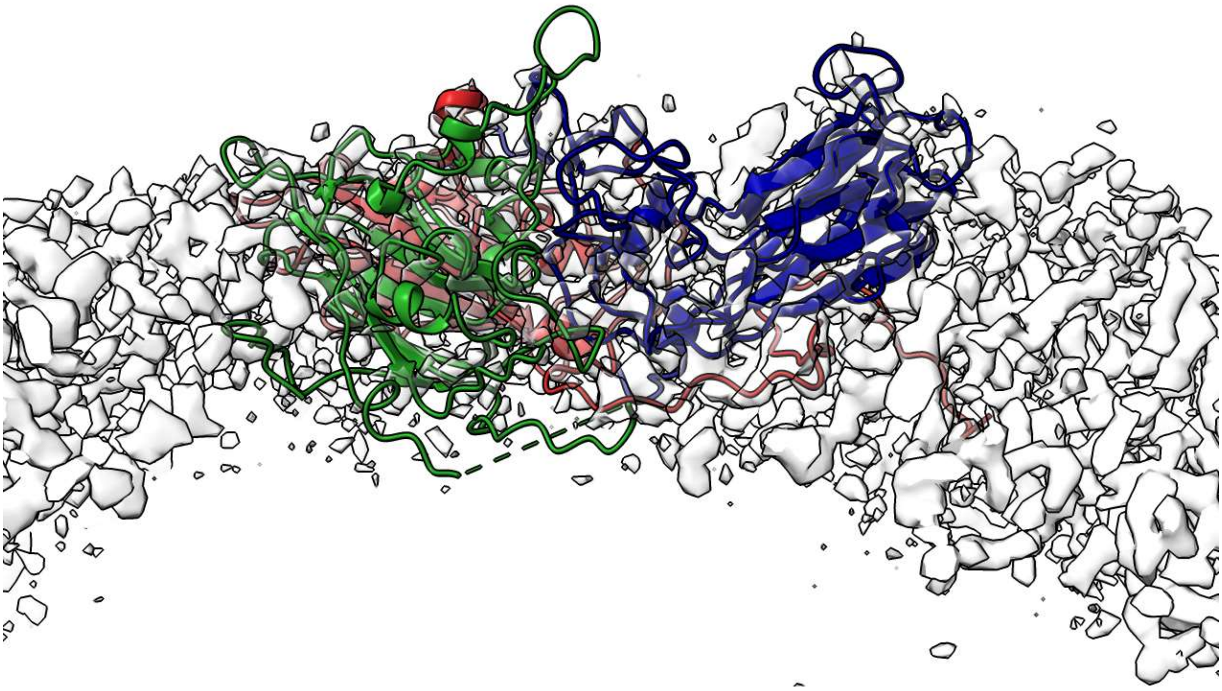

K162E empty

Fig. 19. Details of the side view particle reconstruction. Coulomb-density map corresponding to the side view of WT and K162E particles that were incubated at hig temperature to simulate uncoating. These densities enable model building in which capsid proteins and viral RNA was identified. Each particle class is identified at the bottom of each figure. We were able to identify densities corresponding to pocket factor (purple), surrounded by models of VP1 (blue), VP2 (green), VP3 (red), and VP4 (yellow). While the density for the K162E-native structure is poor, the Coulomb density of the region of the pocket factor cannot be attributed to nearby sidechains.

Movie Legends:

Supplementary Movie 1. Dynamics of the EF and GH loops in VP1 and VP2 of EV-A71 at 30ºC. The movie shows the dynamics of the EF loop in green, the GH loop in cyan,

and the pocket factor in purple during the length of the simulation compared to their initial position, which is maintained fixed during the length of the movie. For clarity, we maintained fixed also the initial position of VP1, VP2, VP3, and VP4 which are represented in white, new cartoon format.

Supplementary Movie 2. Dynamics of the EF and GH loops in VP1 and VP2 of EV-A71 K162E thermostable mutant at 30ºC. The movie shows the dynamics of the EF loop in green, the GH loop in cyan, and the pocket factor in purple during the length of the simulation compared to their initial position, which is maintained fixed during the length of the movie. For clarity, we maintained fixed also the initial position of VP1, VP2, VP3, and VP4 which are represented in white, new cartoon format.

Supplementary Movie 3. Dynamics of the EF and GH loops in VP1 and VP2 of EV-A71 at 52ºC. The movie shows the dynamics of the EF loop in green, the GH loop in cyan, and the pocket factor in purple during the length of the simulation compared to their initial position, which is maintained fixed during the length of the movie. For clarity, we maintained fixed also the initial position of VP1, VP2, VP3, and VP4 which are represented in white, new cartoon format.

Supplementary Movie 4. Dynamics of the EF and GH loops in VP1 and VP2 of EV-A71 K162E thermostable mutant at 52ºC. The movie shows the dynamics of the EF loop in green, the GH loop in cyan, and the pocket factor in purple during the length of the simulation compared to their initial position, which is maintained fixed during the length of the movie. For clarity, we maintained fixed also the initial position of VP1, VP2, VP3, and VP4 which are represented in white, new cartoon format.
