# Supplementary material for: A tradeoff between enterovirus A71 particle stability and cell entry
Source: Nat Commun. 2023 Nov 17;14:7450. doi: 10.1038/s41467-023-43029-0 (PMC10656440; doi:10.1038/s41467-023-43029-0)
Supplement: Supplementary file 2 — Reporting Summary [file 41467_2023_43029_MOESM2_ESM.pdf]

nature portfolio

Corresponding author(s): raul andino

Last updated by author(s): 2023 10 07

Reporting Summary

Nature Portfolio wishes to improve the reproducibility of the work that we publish. This form provides structure for consistency and transparency in reporting. For further information on Nature Portfolio policies, see our Editorial Policies and the Editorial Policy Checklist.

Statistics

For all statistical analyses, confirm that the following items are present in the figure legend, table legend, main text, or Methods section.

n/a	Confirmed	
☐	☒	The exact sample size (n) for each experimental group/condition, given as a discrete number and unit of measurement
☐	☒	A statement on whether measurements were taken from distinct samples or whether the same sample was measured repeatedly
☐	☒	The statistical test(s) used AND whether they are one- or two-sided<br>*Only common tests should be described solely by name; describe more complex techniques in the Methods section.*
☒	☐	A description of all covariates tested
☒	☐	A description of any assumptions or corrections, such as tests of normality and adjustment for multiple comparisons
☒	☐	A full description of the statistical parameters including central tendency (e.g. means) or other basic estimates (e.g. regression coefficient) AND variation (e.g. standard deviation) or associated estimates of uncertainty (e.g. confidence intervals)
☐	☒	For null hypothesis testing, the test statistic (e.g. F, t, r) with confidence intervals, effect sizes, degrees of freedom and P value noted<br>*Give P values as exact values whenever suitable.*
☒	☐	For Bayesian analysis, information on the choice of priors and Markov chain Monte Carlo settings
☒	☐	For hierarchical and complex designs, identification of the appropriate level for tests and full reporting of outcomes
☒	☐	Estimates of effect sizes (e.g. Cohen's d, Pearson's r), indicating how they were calculated

Our web collection on statistics for biologists contains articles on many of the points above.

Software and code

Policy information about availability of computer code

Data collection	SerialEM, NAMD2.14
Data analysis	Relion 3.1, MOTIONCOR2, CTFFIND4, Phenix 1.14, UCSF ChimeraX, CHARMM36m, VMD 1.9.3, MDAnalysis 2.0.0, python 3.7.1, Scipy 1.7

For manuscripts utilizing custom algorithms or software that are central to the research but not yet described in published literature, software must be made available to editors and reviewers. We strongly encourage code deposition in a community repository (e.g. GitHub). See the Nature Portfolio guidelines for submitting code & software for further information.

Data

Policy information about availability of data

All manuscripts must include a data availability statement. This statement should provide the following information, where applicable:

- Accession codes, unique identifiers, or web links for publicly available datasets
- A description of any restrictions on data availability
- For clinical datasets or third party data, please ensure that the statement adheres to our policy

The atomic coordinates of EV-A71 WT Native, WT A-particle, WT Empty, K162E Native, K162E A1-particle, K162E A2-particle, K162E A3-particle, K162E Empty have been submitted to the Protein Data Bank with accession numbers: 8E2X, 8E2Y, 8E31, 8E38, 8E39, 8E3A, 3E3B, and 8E3C, respectively. The cryoEM density maps of EV-A71 WT Native, WT A-particle, WT Empty, K162E Native, K162E A1-particle, K162E A2-particle, K162E A3-particle, K162E Empty have been deposited in the Electron Microscopy Data Bank under accession codes: EMD-27850, EMD-27851, EMD-27853, EMD-27859, EMD-27860, EMD-27861, EMD-27862, and EMD-27863, respectively. Electron micrographs for EV-A71 WT, WT-heated, K162E, and K162E-heated were deposited to the Electron Microscopy Public Image Archive Repository under accessions EMPIAR-11172, EMPIAR-11169, EMPIAR-11174, and EMPIAR-11173.

Field-specific reporting

Please select the one below that is the best fit for your research. If you are not sure, read the appropriate sections before making your selection.

☒ Life sciences ☐ Behavioural & social sciences ☐ Ecological, evolutionary & environmental sciences

For a reference copy of the document with all sections, see nature.com/documents/nr-reporting-summary-flat.pdf

Life sciences study design

All studies must disclose on these points even when the disclosure is negative.

Sample size	No statistical method was used to predetermine sample sizes. Biological triplicates are generally considered acceptable for these types of experiments. The final density maps of EV-A71 WT Native, WT A-particle, WT Empty, K162E Native, K162E A1-particle, K162E A2-particle, K162E A3-particle, and K162E empty were calculated using 20,699, 1,316, 848, 12,966, 2,094, 604, 246, 162 particles, respectively, to provide the overall resolutions of 3.3 Å, 8.0 Å, 14.0 Å, 7.4 Å, 3.1 Å, 7.4 Å, 5.9 Å, and 7.1 Å.
Data exclusions	Micrographs with drift and astigmatism were excluded for further analysis as it usual practice.
Replication	For each experiment technical triplicates were performed, with biological duplicate performed when indicated.
Randomization	Stochastic Gradient Descent was used for de novo 3D inital model and 3D classification.
Blinding	2D and 3D classification of CryoEM structures was performed computationally.

Reporting for specific materials, systems and methods

We require information from authors about some types of materials, experimental systems and methods used in many studies. Here, indicate whether each material, system or method listed is relevant to your study. If you are not sure if a list item applies to your research, read the appropriate section before selecting a response.

Materials & experimental systems

n/a	Involved in the study	
☐	☒	Antibodies
☐	☒	Eukaryotic cell lines
☒	☐	Palaeontology and archaeology
☐	☒	Animals and other organisms
☒	☐	Human research participants
☒	☐	Clinical data
☒	☐	Dual use research of concern

Methods

n/a	Involved in the study	
☒	☐	ChIP-seq
☒	☐	Flow cytometry
☒	☐	MRI-based neuroimaging

Antibodies

Antibodies used	The primary antibody, mouse anti-EV-A71 (Sigma-Aldrich MAB979) was diluted in 5% BSA/TBST. Secondary antibody donkey anti-Goat IgG, HRP (Santa Cruz Biotechnology SC-2020; 1:10,000) was diluted in TBST.
Validation	The antibody against EV-A71 was validated by mock infected cell control and visualization by both immunoblot and immunofluorescence.

Eukaryotic cell lines

Policy information about cell lines

Cell line source(s)	Human Rhabdomyosarcoma (RD) cells used in this study were purchased from ATCC (CCL-136).
Authentication	RD cells were validated by growth and morphology phenotypes in addition.
Mycoplasma contamination	Cell lines were tested prior to propagation and freezing using a PCR assay.
Commonly misidentified lines (See ICLAC register)	No commonly misidentified cell lines were used in this study.

Animals and other organisms

Policy information about studies involving animals; ARRIVE guidelines recommended for reporting animal research

Laboratory animals	We used hSCABR2 transgenic mice provided by Dr. Satoshi Koike. (https://doi.org/10.1073/pnas.1217563110). These mice were bred in the AAALAC certified animal facility in UCSF. 3-wk-old mice were used for i.c. experiment. 4-5 week old mice for i.p. An equal number (or close to 1:1) of male and female mice were used in each experiment.
Wild animals	No wild animals were used.
Field-collected samples	No samples were collected from the field.
Ethics oversight	All animal experiments were conducted in accordance with the guidelines of Laboratory Animal Center of National Institutes of Health. The Institutional Animal Care and Use Committee of the University of California, San Francisco approved all animal protocols (Approved protocol number AN194006-01A).

Note that full information on the approval of the study protocol must also be provided in the manuscript.
