# Supplementary material for: A tradeoff between enterovirus A71 particle stability and cell entry
Source: Nat Commun. 2023 Nov 17;14:7450. doi: 10.1038/s41467-023-43029-0 (PMC10656440; doi:10.1038/s41467-023-43029-0)
Supplement: Supplementary file 3 — Description of Additional Supplementary Files [file 41467_2023_43029_MOESM3_ESM.pdf]

Description of Additional Supplementary Files:
A tradeoff between enterovirus A71 particle stability and cell entry

Adam Catching[1,2], Ming Te Yeh[1], Simone Bianco[3], Sara Capponi[4,5*], Raul Andino[1,*]

[1] Department of Microbiology and Immunology, University of California, San Francisco, San Francisco, CA 94158

[2] Graduate Program in Biophysics, University of California, San Francisco, San Francisco, CA 94158

[3] Altos Labs, Los Altos, CA 94022

[4] Industrial and Applied Genomics, AI and Cognitive Software, IBM Almaden Research Center, San Jose, CA, 95120

[5] Center for Cellular Construction, San Francisco, CA 94158

[*] corresponding authors: sara.capponi@ibm.com; raul.andino@ucsf.edu

Supplementary Movie 1. Dynamics of the EF and GH loops in VP1 and VP2 of EV-A71 at 30°C. The movie shows the dynamics of the EF loop in green, the GH loop in cyan, and the pocket factor in purple during the length of the simulation compared to their initial position, which is maintained fixed during the length of the movie. For clarity, we maintained fixed also the initial position of VP1, VP2, VP3, and VP4 which are represented in white, new cartoon format.

Supplementary Movie 2. Dynamics of the EF and GH loops in VP1 and VP2 of EV-A71 K162E thermostable mutant at 30°C. The movie shows the dynamics of the EF loop in green, the GH loop in cyan, and the pocket factor in purple during the length of the simulation compared to their initial position, which is maintained fixed during the length of the movie. For clarity, we maintained fixed also the initial position of VP1, VP2, VP3, and VP4 which are represented in white, new cartoon format.

Supplementary Movie 3. Dynamics of the EF and GH loops in VP1 and VP2 of EV-A71 at 52°C. The movie shows the dynamics of the EF loop in green, the GH loop in cyan, and the pocket factor in purple during the length of the simulation compared to their initial position, which is maintained fixed during the length of the movie. For clarity, we maintained fixed also the initial position of VP1, VP2, VP3, and VP4 which are represented in white, new cartoon format.

Supplementary Movie 4. Dynamics of the EF and GH loops in VP1 and VP2 of EV-A71 K162E thermostable mutant at 52°C. The movie shows the dynamics of the EF loop in green, the GH loop in cyan, and the pocket factor in purple during the length of the simulation compared to their initial position, which is maintained fixed during the length of the movie. For clarity, we maintained fixed also the initial position of VP1, VP2, VP3, and VP4 which are represented in white, new cartoon format.
